# Drooling may be Associated with Dysphagia Symptoms in Multiple Sclerosis

**DOI:** 10.1007/s00455-024-10666-6

**Published:** 2024-02-19

**Authors:** Merve Sapmaz Atalar, Gençer Genç, Serpil Bulut

**Affiliations:** 1 Department of Speech and Language Therapy, Hamidiye Faculty of Health Sciences, University of Health Sciences, Istanbul, Turkey; 2 Department of Neurology, University of Health Sciences, Şişli Hamidiye Etfal Training and Research Hospital, Istanbul, Turkey

**Keywords:** Multiple sclerosis, Drooling, Dysphagia, Hypersalivation

## Abstract

During the process of the multiple sclerosis (MS), persons with multiple sclerosis (PwMS) may experience drooling (sialorrhea) issues that are frequently disregarded. The exact cause of drooling in PwMS is poorly understood. This study aims to assess potential risk factors for drooling seen in PwMS. The study included 20 PwMS with drooling and 19 PwMS without drooling. The participants’ sociodemographic data and clinical parameters were noted. To evaluate dysphagia, fatigue, and hypersalivation, the Dysphagia in Multiple Sclerosis Questionnaire (DYMUS), the Fatigue Severity Scale (FSS), and objective saliva flow rate measurement with cottons placed in Stensen ducts and under the tongue (swab test) were used, respectively. The study employed univariate and multivariate logistic regression models to identify the risk factors linked to drooling. Gender, age, disease duration, MS type, and Expanded Disability Status Scale scores did not differ between the two groups. There was a significant increase in the DYMUS and submandibular/sublingual (SM/SL) saliva flow rate values in PwMS with drooling (*p* = 0.009 and *p* = 0.019, respectively). However, in our study, hypersalivation was not observed in PwMS with or without drooling. In the univariate model, DYMUS, SM/SL saliva flow rate, and FSS were found to be risk factors for drooling in PwMS. But only DYMUS was shown to be a significant risk factor in the multivariate model obtained by the backward (Wald) elimination method (*p* = 0.023). Finally, our research is the first to demonstrate the relationship between drooling and the presence of dysphagia symptoms in PwMS. This is a very important study to determine the nature of drooling in PwMS. This finding shows that our study will serve as a reference for choosing the best method for drooling treatment.

## Introduction

Multiple sclerosis (MS) is a demyelinating and inflammatory disease of the central nervous system [1]. MS is associated with clinical symptoms, including sensory, motor, and cognitive deficits, such as spasticity, balance and mobility problems, fatigue, pain, vision problems, bladder and bowel dysfunction, sexual dysfunction, depression, and swallowing and communication disorders [2, 3]. Among these symptoms, swallowing disorder is also defined as dysphagia [4]. As in Parkinson’s and Alzheimer’s disease, dysphagia in MS is characterized by disruption of the oral and pharyngeal phases [5–7]. PwMS have various swallowing abnormalities, including impaired tongue movement and reduced tongue base retraction during the oral preparation and transit phase, delayed and prolonged pharyngeal phase, and lack of laryngeal closure [8–11]. Drooling (sialorrhea) can result from difficulties in controlling saliva due to sensory motor impairments in the oral and pharyngeal phases [12]. In this case, drooling can be seen in PwMS and can negatively affect quality of life.

Drooling is the outflow of saliva in the mouth. It is often used interchangeably with the term hypersalivation. However, hypersalivation is defined as excessive saliva production [12]. Saliva is secreted by three main glands: the parotid, SM, and SL glands. The total contribution of the SM/SL glands to the amount of unstimulated saliva is approximately 75%, while the parotid gland contributes approximately 25% [12, 13]. The unstimulated salivary flow rate is approximately 0.3–0.4 ml/min, while values above 0.5 ml/min are considered abnormal [14, 15].

Some of the mechanisms involved in the control of saliva may be related to the mechanisms involved in the oral and pharyngeal phases of swallowing. Processes such as keeping the mouth closed, coordination of tongue movements, and frequency of swallowing may also play a role in the control of saliva. Problems with one or more of the above processes, certain medications, disease severity, disease duration, male gender, and excessive salivation from the salivary glands may be risk factors for drooling [12, 16–18].

The occurrence of drooling in PwMS is often overlooked not only in clinical practice but also in academic research. Although some authors have suggested that dysphagia may be the cause of drooling in PwMS, evidence is needed [19, 20]. Investigating the causes of drooling in PwMS may be important for planning treatment strategies. The aim of this study was to examine possible risk factors, especially dysphagia, for drooling in PwMS. In this way, it is thought that this study will serve as a guide to draw attention to drooling in PwMS, to determine the risk factors for drooling, to help choose the best methods for its treatment, and to fill the gap in the literature.

## Methods

### Study Design and Selection of Participants

The study was carried out in accordance with the Declaration of Helsinki Principles, and informed consent forms were obtained from all participants. The study was approved by the University of Health Sciences XXXX Scientific Research Ethics Committee (22/419). PwMS non-droolers and droolers were evaluated for swallowing problems, saliva flow rate, and sociodemographic and clinical data.

The study was conducted with participants diagnosed with MS according to McDonald criteria in the Neurology Clinic of XXXX and Research Hospital, University of Health Sciences [21]. A total of 19 PwMS non-droolers and 20 PwMS droolers were included in the study. Inclusion criteria for PwMS participating in the study included being over 18 years old, not having a psychiatric diagnosis, not having any additional neurological diseases other than MS, scoring 24 or higher on the Mini Mental State Examination, and not receiving voice, swallowing, or speech therapy within the previous six months. The participants’ oral cavities were examined and they were determined not have gum issues or oral wounds.

The Expanded Disability Status Scale (EDSS) scoring was done by a neurologist. The Drooling Severity and Frequency Scale (DSFS) was completed by PwMS. According to their DSFS ratings, all PwMS who met the study’s inclusion criteria were separated into groups of those who drooled and those who did not. Then, in another room, persons were assessed by a speech-language pathologist by placing and measuring dental rolls for saliva rate. The DYMUS questionnaire and FSS were completed by PwMS.

### Data Collection Tools

#### Demographic Data Collection Form

The form includes the participant’s age, occupation, gender, and medical history. MS type, disease duration, level of independence, use of medications, comorbidities, and prior surgeries are all covered in the medical history section of the form.

#### Mini-Mental State Examination (MMSE)

It is a 30-point test used to evaluate cognitive status. A score of 24 or above indicates normal cognition [22]. A validity and reliability study in Turkey was carried out by Güngen and colleagues [23].

#### Expanded Disability Status Scale (EDSS)

The scale evaluates disability in PwMS, administered in conjunction with interviewing the person, and neurological examination. This scale, which has 20 stages with 0.5 steps between each, ranges from 0 (normal neurological examination) to 10 (MS-related mortality). Functional systems graded in EDSS include visual, brainstem, pyramidal, sensory, cerebellar, bowel and bladder, cerebral, and ambulation [24].

#### Drooling Severity and Frequency Scale (DSFS)

The scale determines the intensity and frequency of salivation by obtaining information from family members or caregivers. Higher scores indicate a worse saliva control status. The severity subscale is scored from 1 to 5, and the frequency subscale is scored from 1 to 4 [25]. In our study, the DSFS score was given as the sum of the severity and frequency scores. Using the DSFS scale, the groups non-droolers and droolers were identified. Non-droolers (without drooling) were those with a DSFS score of 5 or less; droolers (with drooling) were those with a DSFS score of 5 or more [17].

#### Swab Test

To determine whether hypersalivation is the main cause of drooling, a swab test known as the saliva flow rate test was used. It was performed under controlled conditions using the approach described by Erasmus et al. [16]. After the mouth was dried with sterilized gauze, three absorbent rolls of cotton were placed under the tongue for five minutes, one at the opening of the SM/SL gland canals and two at the opening of each parotid canal. We placed SM/SL swab that was across the midline overlying the two papillae of Wharton’s duct. Before the sense of hunger set in and at least one hour after the last meal, the test was conducted while sitting with the head in a neutral position in PwMS. Rolled cotton sponges were measured before and after the procedure with an electronic scale with a precision. Swabs from the parotid papilla were weighed separately from the SM/SL swabs. The resulting increase was translated as gram saliva/min [12, 16].

#### Dysphagia in Multiple Sclerosis Questionnaire (DYMUS)

The scale consists of 10 items specific to MS. DYMUS is an easy-to-apply, reliable, and consistent tool for evaluating oropharyngeal dysphagia in PwMS and identifying its main aspects [26]. All items in the scale are marked as Yes (1) or No (0). The total scale score ranges from 0 to 10. A score of 3 or more on the scale indicates severe dysphagia. A Turkish validity and reliability study was performed by [27] in 2018.

#### Fatigue Severity Scale (FSS)

It is used to evaluate fatigue in PwMS. The scale questions the fatigue status of the individual in the previous month. It consists of nine questions, and each question consists of seven points. High scores indicate fatigue. The validity and reliability studies of the Turkish scale were carried out by Armutlu et al. in 2007 [28, 29].

### Statistical Analysis

IBM SPSS V23 was used to evaluate the data. The Shapiro–Wilk test was used to gauge conformity to the normal distribution. To compare categorical variables between the groups, the Yates-corrected chi-square test was used. The Mann–Whitney U test was used to analyze non-normally distributed data, while the independent two-sample t test was used to compare normally distributed data according to paired groups. To investigate the independent risk factors influencing the existence of drooling, a binary logistic regression analysis was utilized. Two different models, univariate and multiple, were used to examine the effect of independent risk factors on drooling. In the univariate model, the effect of each independent risk factor alone, without other risk factors, was examined. On the other hand, the multiple model is a model created by including more than one risk factor in the model. The results obtained in the multiple models are stronger and more reliable. As a result of the multivariate model created using the backward (Wald) elimination method, cox & Snell R Square 0.264 and Nagelkerke R Square 0.352 were obtained. The correct classification rate was 69.2%. For quantitative data, the analysis results were showed as mean ± standard deviation; for categorical data, they were displayed as frequency and percentage. The level for significance was established at *p* < 0.050.

## Results

24 female and 15 male were participated in the study. Of the 39 participants, 24 were relapsing–remitting MS (RR-MS) and 15 were secondary progressive MS (SP-MS). Participants were divided into 2 groups, non-droolers and droolers. There was no difference between the groups for gender and MS type (*p* = 1.000; *p* = 0.899, respectively) (Table 1).

**Table 1:** Distribution of drooling by MS type and gender

	Drooling		Total (%)	Test statistics	P value
	Non-droolers (%)	Droolers (%)			
Gender					
Female	12 (50)	12 (50)	24 (61.5)	0.000	1.000
Male	7 (46.7)	8 (53.3)	15 (38.5)		
MS Type					
RR-MS	11 (45.8)	13 (54.2)	24 (61.5)	0.016	0.899
SP-MS	8 (53.3)	7 (46.7)	15 (38.5)		
DSFS1 (Mean ± SD)	1.11 ± 0.32	2.55 ± 0.51	1.85 ± 0.84	9.00	** < 0.001**^**b**^
DSFS2 (Mean ± SD)	1.16 ± 0.37	2.65 ± 0.59	1.92 ± 0.90	12.00	** < 0.001**^**b**^
Total DSFS (Mean ± SD)	2.26 ± 0.65	5.20 ± 0.41	3.77 ± 1.58	0.00	** < 0.001**^**b**^

*RRMS* Relapsing–remitting MS, *SP-MS* Secondary progressive MS, *DSFS* Drooling Severity and Frequency Scale, *SD* standard deviation

Significant *p* values are given in bold

^a^Independent two-sample t test

^b^Mann–Whitney U test

A statistically significant difference was found between the total mean DYMUS of the participants according to the presence of drooling (*p* = 0.009). While the mean DYMUS of those non-droolers was 1.42 ± 2.39, the mean of those droolers was 3.65 ± 2.68. In addition, a difference was found between the mean SM/SL total flow rate (gr/min) according to the presence of drooling (*p* = 0.019). While the mean of the non-droolers group was 0.15 ± 0.09, the mean of droolers was 0.24 ± 0.12 (Table 2).

**Table 2:** Comparison of clinical findings, fatigue, dysphagia and saliva flow rate parameters according to the presence of drooling

	Non-droolers	Droolers	Total	Test statistics	*p* value
	Mean ± SD	Mean ± SD	Mean ± SD		
Age	45.47 ± 6.82	47.15 ± 9.69	46.33 ± 8.35	-0.622	0.538^a^
Disease Duration (years)	9.61 ± 8.77	9.35 ± 7.29	9.47 ± 7.94	189.50	0.989^b^
EDSS	3.11 ± 2.95	2.95 ± 2.52	3.03 ± 2.70	180.00	0.792^b^
FSS	42.26 ± 17.49	52.35 ± 7.88	47.44 ± 14.21	133.50	0.113^b^
DYMUS	1.42 ± 2.39	3.65 ± 2.68	2.56 ± 2.75	98.00	**0.009**^**b**^
SM/SL Total Flow Rate (gr/min)	0.15 ± 0.09	0.24 ± 0.12	0.19 ± 0.11	-2.450	**0.019**^**a**^
Right Parotid Flow Rate (gr/min)	0.11 ± 0.08	0.14 ± 0.11	0.13 ± 0.10	152.00	0.296^b^
Left Parotid Flow Rate (gr/min)	0.13 ± 0.11	0.15 ± 0.10	0.14 ± 0.10	160.50	0.411^b^

*EDSS* Expanded Disability Status Scale, *FSS* Fatigue Severity Scale, *DYMUS* Dysphagia in Multiple Sclerosis Questionnaire, *SM/SL* submandibular and sublingual, *SD* standard deviation

Significant *p* values are given in bold

^a^Independent two-sample t-test

^b^Mann–-Whitney U test

Using univariate and multivariate models in binary logistic regression analysis, the risk factors influencing the existence of drooling were investigated. As a result of the univariate model, FSS, SM/SL flow rate (gr/min), and DYMUS were obtained as risk factors. When the FSS score increases by one unit, the risk of drooling increases 1.066 times (*p* = 0.047). When the DYMUS score increases by one unit, the risk of drooling increases 1.434 times (*p* = 0.017). When the SM/SL flow rate increases by one unit, the risk of drooling increases 1.08 times (*p* = 0.031). The multivariate model presents the results obtained by the backward (Wald) elimination method. DYMUS had a substantial effect on the multivariate model (Table 3). Odds ratio (OR) values in Table 3 show how much a unit change in quantitative independent risk factors decreases or increases the risk. The results obtained when the FSS, DYMUS, and Total Flow Rate (gr/min) values are grouped according to the values corresponding to the 25% and 75% ranks are shown in Table 3. As a result of the analysis, a statistically significant effect of 75% rank of the DYMUS parameter was found (*p* = 0.006). The drooling risk of those with a DYMUS score in the 75% rank was 13.2 times higher than those with a DYMUS score in the 25% rank (*p* = 0.006).

**Table 3:** Examination of risk factors affecting the presence of drooling

	Univariate		Multivariate	
	OR (%95 CI)	*p*	OR (%95 CI)	*p*
Gender (Reference: Female)	1.143 (0.314–4.16)	0.839		
Age	1.025 (0.949–1.108)	0.528		
Disease duration	0.996 (0.919–1.079)	0.919		
EDSS	0.978 (0.773–1.238)	0.856		
FSS	1.066 (1.001–1.136)	**0.047**		
DYMUS	1.434 (1.066–1.93)	**0.017**	1.445 (1.053–1.983)	**0.023**
SM/SL total flow rate (gr/min)*	1.08 (1.007–1.159)	**0.031**	1.089 (1–1.186)	0.051
Right parotid flow rate (gr/min)*	1.04 (0.968–1.117)	0.287		
Left parotid flow rate (gr/min)*	1.018 (0.955–1.084)	0.588		
FSS (Reference: ≤ %25 (44))				
%25–75 (44–57)	3.208 (0.628–16.384)	0.161		
≥ %75 (57)	3.5 (0.549–22.304)	0.185		
DYMUS (Reference: ≤ %25 (0))				
%25–75 (0–4)	1.1 (0.192–6.286)	0.915		
≥ %75 (4)	13.2 (2.112–82.5)	0.006		
Total flow rate (gr/min) (Reference: ≤ %25 (11))				
%25–75 (11–24)	1.969 (0.416–9.317)	0.393		
≥ %75 (24)	3.062 (0.539–17.401)	0.207		

Multivariate model in which independent risk factors are included in the model with Backward: Wald method

*EDSS* Expanded Disability Status Scale, *FSS* Fatigue Severity Scale, *DYMUS* Dysphagia in Multiple Sclerosis Questionnaire, *SM/SL* submandibular and sublingual, *OR* Odds ratio, *CI* Confidence interval

Significant *p* values are given in bold

The Cronbach’s alpha coefficient of the DYMUS scale was 0.841, and it was obtained with high reliability. When the item-total correlation coefficients were analyzed, the total correlation coefficient of each item was 0.396 and above, and a positive correlation was obtained between the items and the total scale (Table 4). When it was examined whether there was a connection between drooling and DYMUS, a statistically significant correlation was found between items 3 and 4 (*p* = 0.032 and *p* = 0.020, respectively). There was no statistically significant correlation between drooling and other items of the DYMUS (*p* > 0.005) (Table 5).

**Table 4:** Item analysis and reliability results of the DYMUS

	Mean	SD	Item total correlation	Cronbach’s alpha when item deleted	Cronbach’s alpha
DYMUS 1	0.231	0.427	0.544	0.825	0.841
DYMUS 2	0.359	0.486	0.630	0.817	
DYMUS 3	0.410	0.498	0.481	0.833	
DYMUS 4	0.308	0.468	0.521	0.828	
DYMUS 5	0.231	0.427	0.626	0.818	
DYMUS 6	0.359	0.486	0.630	0.817	
DYMUS 7	0.154	0.366	0.525	0.828	
DYMUS 8	0.231	0.427	0.490	0.830	
DYMUS 9	0.256	0.442	0.576	0.822	
DYMUS 10	0.026	0.160	0.396	0.841	

*DYMUS* Dysphagia in Multiple Sclerosis Questionnaire, *SD* standard deviation

**Table 5:** Comparison of DYMUS items according to groups

	Non-droolers (%)	Droolers (%)	Total	Test statistics	*p*
DYMUS 1					
No	17(89.47)	13(65.00)	30(76.92)	–	0.127**
Yes	2(10.53)	7(35.00)	9(23.08)		
DYMUS 2					
No	15(78.95)	10(50.00)	25(64.10)	2.402	0.121*
Yes	4(21.05)	10(50.00)	14(35.90)		
DYMUS 3					
No	15(78.95)	8(40.00)	23(58.97)	4.605	0.032*
Yes	4(21.05)	12(60.00)	16(41.03)		
DYMUS 4					
No	17(89.47)	10(50.00)	27(69.23)	5.395	0.020*
Yes	2(10.53)	10(50.00)	12(30.77)		
DYMUS 5					
No	16(84.21)	14(70.00)	30(76.92)	–	0.451**
Yes	3(15.79)	6(30.00)	9(23.08)		
DYMUS 6					
No	14(73.68)	11(55.00)	25(64.10)	0.778	0.378*
Yes	5(26.32)	9(45.00)	14(35.90)		
DYMUS 7					
No	17(89.47)	16(80.00)	33(84.62)	–	0.661**
Yes	2(10.53)	4(20.00)	6(15.38)		
DYMUS 8					
No	17(89.47)	13(65.00)	30(76.92)	–	0.127**
Yes	2(10.53)	7(35.00)	9(23.08)		
DYMUS 9					
No	16(84.21)	13(65.00)	29(74.36)	–	0.273**
Yes	3(15.79)	7(35.00)	10(25.64)		
DYMUS 10					
No	19(100.00)	19(95.00)	38(97.44)	–	–
Yes	0(0.00)	1(5.00)	1(2.56)		

Significant *p* values are given in bold

^*^Yates correction, **Fisher’s Exact test

## Discussion

During the course of their illness, drooling, an issue that is frequently ignored, can damage their quality of life for PwMS. However, it is unclear which PwMS may experience drooling and the related risk factors. To reduce the risk of drooling, carry out the required interventions, and improve quality of life, it is crucial to identify risk factors.

There are many factors that can contribute to drooling. However, the affecting factors may differ from disease to disease, and the information in the literature often comes from studies conducted on other neurodegenerative diseases. To the best of our knowledge, there is insufficient information in the literature on drooling in PwMS, and the research is limited. Our study handled the drooling complaints of PwMS using objective and subjective methods and examined various possible risk factors that may cause drooling. Our study emphasizes that drooling may be seen in PwMS and that this may be associated with the presence of dysphagia. It has been reported that the prevalence of dysphagia in PwMS is 45%, and this rate is quite high compared to the general population [30]. In MS, swallowing function may be impaired as a result of lesions in the corticobulbar pathways, cranial nerve paresis, brainstem and cerebellar disorders, and cognitive dysfunction [31, 32]. This may lead to impairment of one or more of the oral, pharyngeal, and esophageal phases. Food being caught in the throat, coughing or choking during or after eating or drinking, the need to swallow multiple times, difficulties starting to swallow, difficulty controlling saliva, drooling, and changes in eating patterns are some of the dysphagia symptoms that PwMS most frequently experience [33].

In our study, no relationship was found between drooling and gender in PwMS. There are different results in the literature regarding the relationship between drooling and gender. Mao et al. [34] reported in their studies with individuals with Parkinson’s disease (IwPD) that drooling was associated with male gender. Kalf et al. [17] reported male gender as a factor explaining drooling in their study with the same disease group. In Erasmus et al. [16] study on children with cerebral palsy, no relationship was found between saliva control problems and gender. The reason for this inconsistency in the results of the studies may be that the studies were conducted with different disease and age groups. Although MS is observed more frequently in women than in men, gender does not seem to make a difference in the development of drooling in MS.

Another factor to take into account when determining how drooling will develop is age. Studies of IwPD have shown that the prevalence of drooling increases with age [17, 35]. In our study, age did not contribute to drooling in PwMS. Unlike Parkinson’s disease, MS often affects a young age group, such as 20–40 years, so it is not surprising that age does not contribute to drooling. The sample included in this study consisted of young adults between 24 and 61 years of age. The absence of older adult PwMS in the sample may explain why age is not a risk factor for drooling.

In our study, no difference was found in MS type, disease duration, and EDSS scores in terms of the occurrence of drooling in PwMS. In fact, we hypothetically predicted that the rate of PwMS with drooling would be higher in persons with secondary progressive MS. However, the results of the two groups did not show a statistically significant difference. We also expected PwMS with drooling to have longer disease durations and higher EDSS scores, although it is not a scale for assessing drooling. This may be due to either the insufficient size of our sample or the presence of other factors affecting drooling, independent of the progression of the PwMS, the duration of the disease, and the level of disability.

There was a significant difference in DYMUS and SL/SM flow rates between the groups with and without drooling. SL/SM flow rate was found to be a risk factor in univariate model. However, although there was a difference between the groups, it was determined that the flow rates observed in both groups were below the limit accepted as hypersalivation. In addition to SM/SL salivary flow rate, FSS values measuring fatigue and the presence of dysphagia were also found to be risk factors for drooling as a result of the univariate model. It should be kept in mind that these factors should also be taken into consideration when evaluating drooling in PwMS.

In the univariate variable model, the risk factors for drooling in PwMS were found to be DYMUS, SM/SL salivary flow rate, and FSS. Each independent risk factor alone had an effect on drooling without other risk factors in the model. Since it is important to see what kinds of results are obtained with the remaining parameters when the variables that have no effect in the model are removed, the backward (Wald) method is preferred. As a result of the multivariate model obtained by the Wald elimination method, only the presence of dysphagia symptoms was found to be a risk factor for drooling. A number of previous studies on various neurodegenerative disorders have shown that drooling and swallowing problems are significantly associated [35–37]. In some studies in the literature, drooling was associated with disruptions in the swallowing phases rather than in the amount of saliva; it was stated that saliva control problems were caused by decreased lip closure or swallowing frequency [12, 38, 39]. The reasons for the differences between the results of the studies may include the inclusion of different disease groups in the studies, the different natures of the diseases, and the different assessment methods.

According to our study, drooling is highly correlated with the presence of dysphagia symptoms in PwMS. This may be because some mechanisms involved in the oral and pharyngeal phases of swallowing are common with drooling. It may be important to evaluate persons’ swallowing phases before planning interventions for drooling in PwMS. The higher incidence of drooling in PwMS with swallowing disorder symptoms in our study indirectly supports these views. However, we cannot conclude that drooling is directly related to the presence of dysphagia because we did not use an instrumental assessment method that could reveal the presence of dysphagia in our study. In our study, we used DYMUS to determine the risk of dysphagia. DYMUS is a scale that actually shows the presence of dysphagia symptoms, not the direct presence of dysphagia.

Since the total correlation coefficient of each item in DYMUS is 0.396 and above, each item was analyzed in our study. A positive correlation was obtained between the items and the total scale. These results show that the scale used is appropriate for the study. A significant correlation was found between the scores of DYMUS items 3 and 4 in the groups with and without drooling. These items include the questions “Do you have a foreign body sensation in your throat while swallowing?” and “Does food stay in your throat?” These items may have a greater impact on saliva control. The fact that the PwMS feels that food remains after swallowing may refer to reduced swallowing efficiency (reduced clearance). This problem may then contribute to a lack of efficiency in swallowing saliva. However, a more detailed dysphagia assessment is needed.

Although we found that drooling was associated with the presence of dysphagia symptoms in PwMS, the presence of drooling in individuals without dysphagia symptoms should also be questioned. In addition, not only should the presence of drooling be questioned but also the ways in which it affects the person (for example, how it affects their life and whether it causes feelings of embarrassment). The use of quality of life scales for drooling will better reveal the impact on persons, especially on social situations [40].

Undoubtedly, our study has some limitations. First, the number of persons in our sample was limited, and all were selected from a tertiary MS reference center. Therefore, it may not be possible to generalize our results to all PwMS. Furthermore, there may be other factors that we did not consider among the factors that may affect drooling. It has been reported in the literature that cognitive impairment may also be associated with drooling [17, 41, 42]. Patient-responsive scales were used in our study. We included persons with good cognitive status to understand and answer the questions of these scales. In future studies, the effects of factors such as drug use, swallowing frequency, or cognitive impairment on drooling can be examined by increasing the sample size. Another limitation of our study is that although we associated drooling with dysphagia in PwMS, we did not examine which symptoms causing dysphagia may be more dominant in drooling. For example, spasticity in PwMS may lead to dysphagia, which in turn may affect drooling. We believe that future research should address the relationship between spasticity, dysphagia, and drooling. However, despite these limitations, our cohort represents a good spectrum of typical PwMS with drooling.

In conclusion, our study is the first to demonstrate the relationship between drooling and dysphagia in PwMS by assessing drooling using both objective and subjective approaches. This finding confirms previous studies showing the association between drooling and dysphagia in neurodegenerative diseases and adds to the literature that this association is present in MS and neurodegenerative diseases. Our study also highlights the need for studies that further examine the importance of drooling and its association with dysphagia in MS and that focus on appropriate interventions to reduce the distressing effects of drooling in PwMS.
